# The SLC6A1 Mutation Schizophrenia case — A Comprehensive Case Study With iPSC Generation

**DOI:** 10.1192/j.eurpsy.2024.1592

**Published:** 2024-08-27

**Authors:** V. Mikhailova, N. Kondratyev, M. Alfimova, V. Kaleda, T. Lezheiko, M. Ublinsky, V. Ushakov, I. Lebedeva, A. Galiakberova, A. Artyuhov, E. Dashinimaev, V. Golimbet

**Affiliations:** ^1^Clinical Genetics Laboratory, Mental Health Research Center; ^2^Department of Radiation Diagnostics, Clinical and Research Institute of Emergency Pediatric Surgery and Trauma; ^3^ Institute for Advanced Brain Research Lomonosov Moscow State University; ^4^N.A. Alekseyev Psychiatric Clinical Hospital No.1; ^5^ National Research Nuclear University MEPhI; ^6^Center for Precision Genome Editing and Genetic Technologies for Biomedicine, Pirogov Russian National Research Medical University; ^7^Faculty of Biology, Lomonosov Moscow State University; ^8^Department of Bioinformatics, Koltzov Institute of Developmental Biology, Russian Academy of Sciences; ^9^Moscow Institute of Physics and Technology (State University), Moscow, Russian Federation

## Abstract

**Introduction:**

The main finding of a large-scale collaborative study (Rees et al. Nat Neurosci 2020;23(2) 179-184), which focused on *de novo* mutations in schizophrenia, was the discovery of an enrichment of these mutations in the *SLC6A1* gene. This gene encodes the gamma-aminobutyric acid (GABA) transporter GAT1, thereby encouraging further research into novel schizophrenia targets within the GABA pathway. However, the gene was not highlighted in recent schizophrenia genetic studies, while typically pathogenic *SLC6A1* mutations result in epilepsy, motor dysfunction, autistic spectrum disorder (ASD) and developmental delay. The absence of genetic replication for *SLC6A1*’s involvement in schizophrenia and the differing clinical spectrum for *SLC6A1* mutations led us to study in depth one of the only three original probands from the Rees et al. 2020 study.

**Objectives:**

In our comprehensive case study, we delved deep into the relationship between the SLC6A1 mutation and schizophrenia.

**Methods:**

Our subject, a patient who first presented with acute mania symptoms at age 15 and was later diagnosed with schizophrenia, carried the SLC6A1 Arg211Cys mutation. Over a detailed 25-year follow-up, we conducted an array of assessments and tests, including cognitive testing, personality assessments, EEG, and 1H-MRS.

**Results:**

Notably, we discovered abnormal GABA levels, potentially indicating a dysfunction in GABA reuptake, adding a new layer of complexity to our understanding. Further analysis revealed a significant correlation between the patient’s clinical picture and a polygenic background, rather than the SLC6A1 mutation. Despite having a high polygenic risk score for bipolar disorder, the dominant features of his condition were more representative of schizophrenia. Interestingly, neither the patient nor his father, who also showed a higher BP PRS, had a diagnosis of bipolar disorder. The pathogenic significance of the mutation warrants investigation in cells of neuronal origin. We generated induced pluripotent stem cells (iPSC) from the patient and his parents. This approach provides us with a platform for future investigations into the pathogenic significance of the mutation in neuronal cells. The Human Pluripotent Stem Cell Registry accession numbers of those cells are MHRCCGi001-A (patient), MHRCCGi005-A (mother) and MHRCCGi004-A (father).

**Conclusions:**

In the presented case the clinical picture is rather explained by the polygenic background than by the SLC6A1 Arg211Cys mutation. The study is supported by Russian Science Foundation, grant 21-15-00124 (https://rscf.ru/project/21-15-00124)

**Disclosure of Interest:**

None Declared

